# Healthcare professionals’ and patients’ views and experiences of surgical and medical treatment for nasal obstruction: a qualitative interview study for a Nasal Airway Obstruction Study (NAIROS)

**DOI:** 10.1136/bmjopen-2025-099395

**Published:** 2025-06-08

**Authors:** Kelly E Lloyd, Caroline Wilson, Sean Carrie, Leila Rooshenas, Janet A Wilson, David Hamilton, Emma Clark, Nikki Rousseau

**Affiliations:** 1 Leeds Institute of Health Sciences, University of Leeds, Leeds, UK; 2 Northern Ireland Clinical Trials, Belfast, UK; 3 Freeman Hospital, Newcastle Upon Tyne Hospitals NHS Foundation Trust, Newcastle upon Tyne, UK; 4 School of Population Health Sciences, University of Bristol, Bristol, UK; 5 Population Health Sciences Institute, Newcastle University, Newcastle upon Tyne, UK; 6 Newcastle Clinical Trials Unit, Newcastle University, Newcastle upon Tyne, UK; 7 Leeds Institute of Clinical Trials Research, University of Leeds, Leeds, UK

**Keywords:** Head & neck surgery, OTOLARYNGOLOGY, QUALITATIVE RESEARCH, Head & neck surgery

## Abstract

**Objectives:**

To understand healthcare professionals’ and patients’ views and experiences of septoplasty and medical management (ie, nasal steroid and saline sprays) for nasal obstruction.

**Design:**

Nested qualitative study as part of the Nasal Airway Obstruction Study (NAIROS) trial. We used in-depth interviews to develop a coding framework based on thematic analysis.

**Setting:**

NAIROS was a trial based in the UK from January 2018 to December 2020 that aimed to compare the effectiveness of septoplasty versus medical management.

**Participants:**

We purposively sampled and interviewed 14 healthcare professionals (surgeons, research nurses) and 31 patients involved in the NAIROS trial across 14 UK hospital sites.

**Results:**

In usual practice, surgeons’ decisions regarding treatment for nasal obstruction are based on a complex assessment of patients’ symptoms, history and anatomy. Surgeons viewed septoplasty as a complex although routine operation, which is not guaranteed to improve symptoms of nasal obstruction. Some patients saw septoplasty, intuitively, as a ‘fix’ for a bent septum, whereas others were keen to avoid surgery if possible. Healthcare professionals welcomed the increased use of standard measurements if these were shown to provide a reliable guide to patient outcomes. However, they felt that it was important to retain an element of clinical judgement. Despite generally good outcomes from septoplasty, some patients still felt they had received little to no benefit from the operation. Patients also reported being underprepared for postsurgery recovery. Experiences were more varied with medical management, with some experiencing symptom improvement, but others discontinuing treatment due to difficulty or pain using the sprays, or perceived ineffectiveness. Remembering to use the sprays could be perceived as burdensome, although most patients were able to incorporate this into their daily routines.

**Conclusions:**

Our qualitative study demonstrated varied individual experiences among patients undergoing septoplasty and medical management. Surgeons welcomed more standard measurements to guide decision-making for septoplasty. For patients, better information about treatment mechanisms, treatment delivery and aftercare, and the development of decision support tools would enable shared decision-making and help to provide optimal patient experience of the treatments.

**Trial registration number:**

ISRCTN16168569.

**Strengths and limitations of this study**

- We conducted a qualitative interview study that allowed us to explore in-depth the experiences of septoplasty and nasal obstruction among both healthcare professionals and patients.
- We interviewed healthcare professionals and patients from multiple hospitals, and patients from a range of age groups.
- However, the patient sample was less diverse in terms of ethnicity, with a majority white sample.
- We explored experiences of treatment of nasal obstruction among a sample recruited to a clinical trial, which may affect generalisability of the results to routine care.

## Introduction

 Septoplasty is a surgical procedure to straighten the nasal septum and is often performed in conjunction with turbinate reduction.^1^ Septoplasty is offered to patients who are experiencing feelings of nasal obstruction and associated symptoms such as disturbed sleep, pronounced snoring and olfactory dysfunction.^2^ Previous quantitative research suggests that a moderate to high number of patients (56%–100%) undergoing septoplasty are satisfied with the outcome.^2 3^ However, outcomes of septoplasty on symptoms and quality of life may be more mixed,^4 5^ and patients can experience adverse effects following the operation, such as bleeding, facial pains, septal adhesions and perforation.^6^ Septoplasty is currently considered a criteria-based access operation across multiple National Health Service hospitals. However, these criteria involve the use of subjective interpretation, and it is unknown how surgeons make decisions about the suitability of patients for septoplasty.

Medical management can also be prescribed for patients experiencing nasal obstruction symptoms. The nasal lining, particularly the turbinates, can become swollen due to allergies or infection leading to nasal obstruction,^7^ which steroid sprays may be able to treat. Medical management has the potential to reduce the number of unnecessary septoplasties for patients who would instead experience symptom relief from sprays. However, patients may also experience adverse effects from the sprays, such as nasal dryness, irritation and bleeding.^8^ To our knowledge, no studies have investigated qualitatively patient or health professional experiences of delivering and receiving septoplasty compared with medical management.

Nasal Airway Obstruction Study (NAIROS) was a randomised controlled trial, which aimed to compare the effectiveness of septoplasty, with or without turbinate reduction, against the use of medical management using a nasal steroid and saline spray.^9^ The trial was conducted across 17 secondary care hospital trusts in England, Scotland and Wales, with the locations listed in the full trial report.^10^ Patients were randomised to either septoplasty or daily nasal steroid and saline sprays.^9^ The trial concluded that septoplasty was more effective at improving patients’ sinonasal symptoms than medical management.^11^ A qualitative nested study was also conducted alongside the trial,^10 12^ which aimed to describe healthcare professionals’ and patients’ experiences of the NAIROS trial, trial recruitment, the interventions under evaluation, and to identify barriers to the implementation of the trial findings. In this paper, we are reporting on healthcare professionals’ and patients’ views on septoplasty and medical management for the treatment of nasal obstruction, and their experiences of these treatments.

## Methods

### Design

We conducted a nested qualitative study to explore the experiences of patients and clinicians participating in the NAIROS trial. We aimed to capture healthcare professionals’ and patients’ views and decision-making on septoplasty and medical management, and their experiences of delivering or undergoing these treatments. The NAIROS protocol was previously published.^9^ Details of the qualitative study are provided below. Standards for Reporting Qualitative Research guidelines for reporting qualitative research were followed throughout.^13^

### Participants and recruitment

At trial recruitment, we invited eligible patients who had agreed or declined to participate in the trial to consent to be contacted by the NAIROS qualitative substudy team. Participants were subsequently purposively sampled (site, gender and allocated arm) and approached using their preferred method of contact (telephone or email), with further information and invited to take part in an in-depth telephone interview. A further purposive sample (site, gender, allocated arm and participants discontinuing allocated arm) was recruited to a follow-up interview—this included but was not limited to people who had participated in an initial interview. We also invited a purposive sample (site, role) of healthcare professionals (surgeons, research nurses) involved in the NAIROS trial to participate in an interview.

We interviewed patients mainly at two time points: recruitment (to understand views and experiences of nasal obstruction) and approximately 6-month follow-up (to understand experiences of surgical and medical treatment for nasal obstruction). A small number of additional interviews were conducted at other time points to understand patients’ reasons for discontinuing their allocated treatment. Patient interviews were conducted between February 2018 and January 2020. NAIROS staff members were interviewed between November 2019 and February 2020 (at the end of trial recruitment—to gather information about experiences of the trial and the delivery of surgical and medical management).

### Interview processes

We developed the topic guides (online supplemental materials) after reviewing research relating to trial recruitment and conduct,^14 15^ septoplasty^2 3^ and after discussions with the wider NAIROS team. The topic guide was updated during the study on the basis of early interviews and participation in the NAIROS trial management group. Interviews were conducted by three experienced qualitative researchers (JM, LL and CW). NR, JM, LL and CW were involved in the nested qualitative study and were part of the NAIROS trial team and attended the trial management group meetings.

### Data management and analysis

We audio-recorded and transcribed verbatim all interviews, and transcripts were managed in NVivo (V.1.2). During data collection, regular meetings were held (NR and JM/CW) to review transcripts, discuss the preliminary findings and to make decisions regarding further data collection.^16^ Recruitment ceased when it was agreed at these meetings that we had collected rich and comprehensive data regarding the topics under investigation, that recent data collection was adding relatively few new insights, and that further data collection was unlikely to add substantially to the analysis, based on the preliminary analysis and with reference to the purposive criteria (data saturation).^17^ To analyse the data, we used thematic analysis with a coding framework.^18 19^ Two researchers (NR and KEL) developed the framework after reading through several transcripts and after reviewing previous literature on qualitative research in trials.^20^ A single coding framework was developed for both patient and staff interviews (online supplemental materials), as many codes were deemed to be relevant to both participant groups (eg, both groups described their experiences of septoplasty and medical management). One researcher (KEL) coded all staff and patient interviews using this single coding framework. KEL joined the qualitative team during trial follow-up and analysis and was not part of the NAIROS trial team. Two authors (KEL and NR) discussed the codes collaboratively and generated themes through discussions.

### Patient and public involvement

Patient and public involvement representatives were involved in the development of the NAIROS trial and contributed to the qualitative design (ie, recruitment strategy, study materials).

## Results

We interviewed 14 NAIROS staff members (table 1), and interviews lasted between 24 and 81 min. 39 patient interviews were conducted with 31 patients (table 2), and interviews were between 6 and 33 min in duration. We identified two main overarching themes: (1) decision-making regarding treatment and (2) experiences of treatment for nasal obstruction.

**Table 1:** Description of the staff interviewed in NAIROS (n=14)

	n
Site number	
1	1
2	3
3	1
4	1
5	2
6	1
7	1
8	1
9	1
10	1
11	1
Profession	
Surgeon	9
Research nurse	5

NAIROS, Nasal Airway Obstruction Study.

**Table 2:** Description of the patients interviewed in NAIROS (n=31)

	n
Intervention	
Septoplasty	13
Medical management	16
N/A—dropped out of trial	1
N/A—declined the trial	1
Gender	
Male	21
Female	10
Ethnicity	
White	28
Asian	1
Other ethnicity	2
Age	
18–30	9
31–40	6
41–50	5
51–60	5
61–70	4
70+	2
Site number	
1	1
2	10
3	2
5	5
6	2
8	1
10	3
11	2
12	2
13	2
14	1
Number of interviews	
Recruitment interview only	17
Post treatment interview only	6
Recruitment and post treatment interview(s)	7
Declined the trial interview	1

NAIROS, Nasal Airway Obstruction Study.

### Decision-making regarding treatment

There was considerable complexity and heterogeneity in terms of both patients’ symptoms of nasal obstruction and how clinicians used these symptoms, together with other factors, to make a decision about whether or not to offer surgery. Staff described a decision-making process (outside the NAIROS trial) which took into account the nature and severity of the patients’ symptoms; the length of time these had been experienced; the nature of symptom onset (whether it was associated with a particular injury or other event); whether symptoms varied seasonally; and the structure of the nose, including the degree of deviation and where the deviation occurred.

In terms of assessing patients it was really the impact that it was having on their quality of life, quality of sleep, relationships with others, the severity of their nasal septal deflection, other possible causes of a blocked nose…So the decision to operate was not really based on just the degree of deflection of the septum, it was the additional symptoms as well. Surgeon 7

Both patients and staff reported that nasal obstruction resulted in a wide range of symptoms. Patients described presenting with multiple different symptoms in clinic including nasal blockage, trouble sleeping and frequent nosebleeds (table 3). Several patients could recall an injury that had damaged or broken their nose, while others did not remember such an incident. Staff members also reported a complex pattern and history of symptoms presented by patients in clinic.

**Table 3:** Patients’ descriptions of their symptoms which led to them being referred to the ENT clinic

Patients’ symptoms	Example from patient
Feelings of nasal blockage	“My right nostril is pretty much useless, it’s blocked 99% of the time.”Patient 1
Difficulties sleeping	“More recently, it had got to the stage where it was causing me a lot of difficulty at night sleeping.”Patient 2
Snoring	“I started snoring and I’d never snored in my life, and obviously it’s waking me up in the middle of the night, you know?”Patient 3
Dry mouth	“And my nose would just shut down, and I’d end up breathing through my mouth, and wake up in the middle of the night with a mouth that’s like sandpaper.”Patient 2
Frequent nosebleeds	“I had frequent nosebleeds throughout the day; I could just randomly have one without any trauma to the nose.”Patient 4
Recurrent sinus infections and migraines	“I was constantly getting sinus infections. I was very prone to sinus infections. I used to get a lot of headaches.”Patient 5
Olfactory dysfunction	“It is not that good because I can’t really smell food. The past year I haven’t been able to smell food properly.”Patient 6
Difficulties exercising	“When I go running, or if I do exercise, then it’s slightly harder than what I remember.”Patient 7

ENT, ear, nose and throat.

I suppose there are the people that are not quite congenital, but something has happened very early in life and they have grown up with it to some extent. […] Sometimes it’s because they have developed a bit of an allergy or something, and maybe the septum is not the whole story.

Then I suppose quite a few of them are traumatic, and that can be people who play rugby or football or things like that, […] Then you probably move on to older people, who I think have probably always had a bit of a septal deviation, but the cartilages have been quite firm. Then they get to 50, 60 or a bit more […] and then they have a problem. Surgeon 4

The heterogeneity across patients seen in clinic and the lack of standardised measurement tools for assessing patients’ septum and symptoms has led to a subjective approach among clinicians with regard to septoplasty referrals.

Examining the nose, I will be looking to see if they actually do have a septal deviation and if, in my mind, I think it’s significant enough to cause the degree of symptoms that they have. If they have such a mild deviation and such a significant degree of symptoms, I wouldn’t necessarily correlate the two. Surgeon 6

Clinicians also displayed subjective decision-making when managing patients’ symptoms. Most surgeons reported recommending medical management to patients first before offering surgery. However, some clinicians stated that in routine care they would often refer patients straight for surgery if their septum was perceived to be highly deviated, or if they had a particular incidence of a traumatic injury.

I’ve always maintained giving them proper medical treatment first. Surgeon 6

Interviewer: And in terms of the reasons why you would put people straight through to surgery?

Participant: Well might be the GP letter, it might be the patient’s history, you know, I could breathe alright up my nose then I fell on it […] And just the severity of some people [who] have a very twisted septum […] so those ones you’d be more inclined just to go for surgery. Surgeon 8

While surgeons often recommended medical management first, some patients interviewed described how they could not always see the value of medical management first, as they perceived the cause of their symptoms to be from a traumatic injury that only surgery could ‘fix’. While they understood the mechanism of action for surgical intervention, they lacked a comparable mechanism of action for medical management, with the use of sprays appearing incompatible with the explanatory model held by these patients for their symptoms.

My first reaction, to be honest, I told her that I would like to have an operation done because, in my personal opinion, if the bone is then tilted towards the side, a medicine cannot cure that. Patient 16

Because I was put in this situation with a traumatic blow to the face, I think, probably, going under the knife was the way to fix it. Patient 18

Other patients interviewed were uncertain as to the best treatment for their nasal symptoms and understood that surgery may not relieve these symptoms. These patients welcomed the opportunity to start on medical management first before surgery.

A friend of mine, he’s just had, basically, the same surgical procedure done, and it’s made no difference to him whatsoever. Patient 19

I think people think of an operation as a straightforward thing, but nothing is straightforward, […] And with the success rates, as well, they weren’t 100% that it would work. So, I thought well, rather than going down the route of the operation, why not try the study, using the spray, and see what sort of success I have from that first. Patient 20

The NAIROS trial incorporated objective (eg, rhinospirometry) and patient-reported measures within the baseline assessments. Several surgeons felt that tools such as these had a potential role in assessing which patients will benefit from septoplasty and which from medical management. However, one surgeon was cautious about relying on standardised measures for septoplasty referral.

If we find that some of the symptoms scores have a good correlation with people that benefit, or the rhinospirometry measurements and stuff correlate well, then that might be quite a useful way of judging who would be a good candidate for the operation and who might not. Surgeon 4

I’m not convinced it’s [assessment tools] the only thing that you should rely on. Surgeon 5

### Experiences of treatment for nasal obstruction

This theme relates to the experiences of delivering (staff) and receiving (patients) treatment for nasal obstruction.

#### Performing septoplasty: variation in surgical practice

Surgeons reported that septoplasty was an operation that they had learnt to perform independently relatively early in their ear, nose and throat (ENT) surgical career. Septoplasty was described as a commonly occurring operation in ENT, but also as a procedure that can be difficult to accomplish well.

It’s a slightly odd operation in that it’s one that we would normally, traditionally, say, ‘That’s a junior’s, registrar’s, operation.’ […] Actually, I think it can be quite a tricky operation to get right. Surgeon 2

Variation in practice was not only limited to the criteria for septoplasty referrals. There was also variation in how surgeons carried out septoplasty, with one surgeon describing that their surgical technique changed as they gained more experience carrying out the operation.

The subtleties, as you gain more experience, you realise it’s not just the septum which causes problems with nasal airflow, there is also the soft tissue and the cartilages in particular, the lateral cartilages which can also have an effect, and it’s identifying those and addressing those subtleties which give a better outcome for the patients. Surgeon 3

Another key point of variation among surgical practice was the surgeon’s threshold for offering turbinate reduction alongside septoplasty.

So for me, I think I have a relatively low threshold for offering turbinate reduction at the time of septal surgery. Patients are clearly having a general anaesthetic anyway to correct the septum, reducing the bulk of the turbinate in my opinion is going to lead an improvement in the […] airway. Surgeon 7

#### Healthcare professionals’ views on medical management changed following participation in the NAIROS trial

One research nurse stated that when they originally took part in the NAIROS trial, they felt that the aim of the trial was to confirm the effectiveness of septoplasty. However, after observing that patients returning for their NAIROS review appointment had positive and negative experiences of both treatment arms, they changed their view and instead saw it as a trial comparing two equal treatments.

Interviewer: “Did you have any reservations about NAIROS before you started recruiting to it?”

Research nurse 1: “Yes, because with thinking that it was just to confirm what people thought I, kind of, had the reservation thinking that people who were randomised to the medical arm were not getting the optimum treatment.”

Interviewer: “Right. Did that change, then, in terms of …?”

Research nurse 1: “Yes, when people have come back and people have been quite happy with the sprays.”

Similar to the research nurse, this surgeon felt that their views on medical management had changed as a result of participating in NAIROS, although they remained sceptical as to whether it was a viable long-term solution for patients.

Yeah I think they have [views on medical management have changed]. I think in some respect I’ve been surprised about some patients being quite positive about it. … I think on balance for the 12 months that they’re taking the medication, some of them have certainly found it useful. I’m not sure how that would play out if we’d sort of monitored them all over five years or so. Surgeon 7

Whether this change in view of the effectiveness of medical management would result in changes in practice was less clear. Most surgeons reported that they already often recommended steroid sprays to patients before offering surgery. However, routine recommendation of saline sprays had been relatively uncommon prior to NAIROS, with one surgeon reporting that as a result of their participation in the NAIROS trial, they now recommended the use of saline sprays to patients.

Personally, I don’t think it’s changed what I do. I’ve always maintained giving them proper medical treatment first. Surgeon 6

I didn’t tend to use the saline douching as much as the protocol did […]. I tend to now, because I sort of got used to the study protocol and I think, ‘Yes, that makes some sense’. Surgeon 2

#### Patients’ experiences of treatment

##### Septoplasty

We explored the experiences of patients randomised to septoplasty. Typically, these patients described the operation as a neutral or positive experience. However, some patients encountered painful side effects following surgery.

Yes, surgery was fine, all well-handled, everything was explained to me again, and I have no adverse comment whatsoever, everything went well. Patient 8

Well, I was in a lot of pain. Pretty much every day was just constant dripping from my nose afterwards. Patient 10

Several patients desired further information on recovery and after-care following surgery. These patients felt uninformed following their operation, such as what they could or could not do during recovery.

To be honest with you, I got no information at all after the operation. I was told to do the nasal washouts and that was pretty much it, but I got no other information. When I rang the hospital about it, all I got was, ‘Well, yes, it’s just a water solution that you're supposed to be using.’ That was it. Patient 10

Patients also described varying levels of success with septoplasty for managing their symptoms. However, compared with medical management, those who found the operation effective typically experienced quite high levels of success, with patients feeling they had experienced a noticeable reduction in their symptoms following the operation.

So, you know, ultimately, having the septoplasty, although it was uncomfortable and not particularly pleasant, it has been great. I can now breathe. Patient 12

I’m still having trouble sleeping, but … I don’t think it’s down to my nose. In fact, my breathing is a lot better now. Patient 13

However, a small number of patients who underwent septoplasty felt the operation resulted in little to no change in their symptoms.

The surgery itself was fine. The results I'm not particularly happy about because I'm feeling no change at all in the way I'm breathing. Patient 1

##### Medical management

For patients in the medical management group, remembering to use the sprays could be burdensome. Patients sometimes described establishing a routine to help them remember, for example, placing the bottle next to their toothpaste.

Using them has been no problem at all. I think it’s remembering when to use them and the frequencies to use. Initially, I had to keep checking on the packaging and what have you. But, as time has gone on, I’ve found it a lot easier, and it’s one of the first—I just do it in the morning now, so I know exactly when I’m having it. Patient 20

There was a lack of clear information on how patients should administer the sprays to an obstructed nasal passage, which resulted in some patients experiencing side effects, such as nosebleeds and pain. One participant reported being advised that their nosebleeds may have been associated with the mode of administration (eg, inserting the spray bottle too deeply), and other people highlighted that using the spray was difficult when there was an obstruction.

To be honest, [the nasal sprays] didn’t do good for me at all…No, it sort of gave me two nosebleeds. I've never experienced nosebleeds before, so I didn't get on well with them at all. Patient 8

Patients also reported varying levels of success with medical management for treating their symptoms. Some perceived no beneficial effects and/or marginal benefits that were outweighed by the disadvantages of use (eg, pain, nose bleeds). These patients typically rapidly discontinued use and sought surgical intervention. Other patients were more convinced that the spray offered benefits, although often small and/or short-lived.

They asked me if I'd want to carry on for a little bit longer with the nasal spray, so I tried for another week, but it was just too sore. […] I didn't really see any changes at all, not for the better and not for the worse either. Patient 1

With the medication I'm taking now, it will give me temporary, two or three hours, relief and that’s it. Patient 11

One person reported significant improvement with the sprays and was informed at their clinical review that their enlarged turbinate had shrunk as a result of treatment, providing a clear mechanism to explain the improvement experienced.

Interviewer: “How has your breathing been since you started on the sprays?”

Patient: “Actually, I was waiting for this question to come up. Much better, but I still get a little bit of congestion. I didn’t really have any side effects; it really helped me. […] When I went to see the consultant at the end, she said that, I think it was turbinate up my nose or something had been quite swollen, but I think the steroids had shrunk them a bit. So it seemed to really have cleared up my airways.” Patient 21

## Discussion

Our nested qualitative study conducted as part of the NAIROS trial identified considerable variation and complexity in decision-making about septoplasty. This included deciding which patients should be offered surgery in the place of medical management, when they should be offered surgery and how surgery was conducted. Furthermore, patients presented with a diversity of symptoms in clinics. Surgeons noted the lack of information to guide decision-making and welcomed a potential place for the use of standardised measures in their usual practice, provided these measures were shown to be a useful guide to patients’ outcomes. However, it was also felt that these measures might not cover all relevant factors. Patients could not always see the value of using medical management first before septoplasty, as only surgery was viewed as a ‘fix’ for a bent septum. In contrast, others were keen to try the sprays to avoid surgery where possible.

In our interview study, we observed differing levels of symptom resolution of nasal obstruction among patients in both medical and surgical management arms, following receipt of their allocated treatment. However, many patients interviewed who underwent septoplasty reported experiencing symptom reduction, which is aligned with the NAIROS clinical trial results.^11^ The trial concluded with a recommendation that people presenting with nasal obstruction symptoms associated with a deviated septum, with an absence of coexistent nasal/sinus disease and with a baseline score of >30 on the Nasal Obstruction Symptom Evaluation (NOSE) scale,^21^ should be offered septoplasty.^11^ The secondary trial analysis also found that patients with a NOSE score indicating more severe nasal obstruction experienced greater benefit from treatment as compared with those with moderate scores. These findings and the use of the standardised NOSE scale should help to support surgeons in their decision-making as to whether to offer septoplasty to patients. It is important to note, however, that several patients described experiencing some level of symptom relief from using the steroid and saline spray, and many were open to trying medical management first before surgery. This generally aligned with the views of healthcare professionals, many of whom already offered a trial of medical management prior to an offer of surgery. Some healthcare professionals also changed their view of, or approach to, medical management following their experiences with the NAIROS trial.

Despite the benefits of septoplasty,^11^ a few patients felt the operation resulted in minimal change to their symptoms, and several experienced unpleasant side effects following surgery. These findings are aligned with previous quantitative research that obtained mixed findings in relation to patients’ reported rates of satisfaction and quality of life following septoplasty.^2^,^5^ In our study, several patients suggested that information on recovery postseptoplasty was lacking, which led to them feeling unsupported following surgery. Inadequate information on aspects such as recovery has been linked to dissatisfaction with surgery and increased anxiety levels among those undergoing day surgery.^22^ More comprehensive and standardised information on recovery postseptoplasty might increase patients’ feelings of support and satisfaction with surgery. In addition, several patients randomised to medical management described a lack of clear information on how to administer the sprays to an obstructed nasal passage, which may have contributed to a lack of effect and side effects such as nose bleeds. Where surgeons feel that a trial of medical management is warranted, providing a clear mechanism of action for the spray and guidance on using the sprays safely, taking into account any septal deviation, may improve adherence and patient satisfaction.

Overall, our findings demonstrate that decision-making on whether to offer patients surgery or medical management is complex, and there are side effects to both treatments. While some patients achieve substantial symptom relief with septoplasty, this is not the case for everyone. It is important, therefore, for support tools, such as decision aids, to be developed. Decision aids have previously been found to support patients’ decisions on surgical treatments, such as by increasing patients’ knowledge on the surgical procedure while decreasing decisional conflict.^23^ Future research should be directed towards developing a patient decision aid for septoplasty, to support informed and shared decisions among patients and their surgeons.

### Strengths and limitations

A strength of the study is that we interviewed patients from multiple hospitals and from a range of age groups. Additionally, we interviewed surgeons and research nurses from multiple hospitals. A limitation is that we interviewed staff and patients recruited to a clinical trial, which may create difficulties when generalising the findings to routine care. In particular, patients who took part in the trial may be more motivated to engage with and adhere to the sprays, compared with those typically seen in routine care. While we aimed to explore decision-making on septoplasty and medical management, participants were randomly assigned to their treatment as part of the NAIROS trial. However, we also explored participants’ wider views, including surgeons’ decision-making on septoplasty in routine care. The patient sample was also limited on certain demographics, with a majority white sample. This is important because there is evidence of variation in the frequency of surgery for nasal obstruction across different ethnic groups, with people who identify as Chinese or black less likely to undergo this surgery than people who identify as white.^24^ Further research is needed to understand this variation.

## Conclusions

Overall, we found that surgeons’ decisions regarding the appropriateness of surgery for individual patients were made on the basis of a complex and largely subjective combination of symptoms, history and patient anatomy. Surgeons indicated that they would welcome clearer criteria to guide decision-making. Several patients reported being under-prepared for postseptoplasty recovery and for using the medical management sprays with an obstructed nasal passage. Better information and the development of decision aids could help to support shared decision-making among surgeons and patients. In turn, this may help to improve patients’ experiences of treatment for nasal obstruction.

## Supplementary material

10.1136/bmjopen-2025-099395 online supplemental file 1

10.1136/bmjopen-2025-099395 online supplemental file 2

10.1136/bmjopen-2025-099395 online supplemental file 3

## Data Availability

Data are available on reasonable request.

## References

[1] van Egmond MMHT, Rovers MM, Tillema AHJ (2018). Septoplasty for nasal obstruction due to a deviated nasal septum in adults: a systematic review. Rhinology.

[2] Tsang CLN, Nguyen T, Sivesind T (2018). Long-term patient-related outcome measures of septoplasty: a systematic review. Eur Arch Otorhinolaryngol.

[3] Toyserkani NM, Frisch T (2012). Are too many septal deviations operated on? A retrospective patient's satisfaction questionnaire with 11 years follow-up. Rhinology.

[4] Valsamidis K, Printza A, Constantinidis J (2020). The Impact of Olfactory Dysfunction on the Psychological Status and Quality of Life of Patients with Nasal Obstruction and Septal Deviation. Int Arch Otorhinolaryngol.

[5] Hytönen ML, Lilja M, Mäkitie AA (2012). Does septoplasty enhance the quality of life in patients?. Eur Arch Otorhinolaryngol.

[6] Arunachalam PS, Kitcher E, Gray J (2001). Nasal septal surgery: evaluation of symptomatic and general health outcomes. Clin Otolaryngol Allied Sci.

[7] Skoner DP (2001). Allergic rhinitis: definition, epidemiology, pathophysiology, detection, and diagnosis. J Allergy Clin Immunol.

[8] Donaldson AM, Choby G, Kim DH (2020). Intranasal Corticosteroid Therapy: Systematic Review and Meta-analysis of Reported Safety and Adverse Effects in Adults. Otolaryngol Head Neck Surg.

[9] Rennie KJ, O’Hara J, Rousseau N (2020). Nasal Airway Obstruction Study (NAIROS): a phase III, open-label, mixed-methods, multicentre randomised controlled trial of septoplasty versus medical management of a septal deviation with nasal obstruction. Trials.

[10] Carrie S, Fouweather T, Homer T (2024). Effectiveness of septoplasty compared to medical management in adults with obstruction associated with a deviated nasal septum: the NAIROS RCT. Health Technol Assess.

[11] Carrie S, O’Hara J, Fouweather T (2023). Clinical effectiveness of septoplasty versus medical management for nasal airways obstruction: multicentre, open label, randomised controlled trial. BMJ.

[12] Moore GF, Audrey S, Barker M (2015). Process evaluation of complex interventions: Medical Research Council guidance. BMJ.

[13] O’Brien BC, Harris IB, Beckman TJ (2014). Standards for reporting qualitative research: a synthesis of recommendations. Acad Med.

[14] Murray E, Treweek S, Pope C (2010). Normalisation process theory: a framework for developing, evaluating and implementing complex interventions. BMC Med.

[15] May CR, Cummings A, Girling M (2018). Using Normalization Process Theory in feasibility studies and process evaluations of complex healthcare interventions: a systematic review. Implement Sci.

[16] Grbich C (1998). Qualitative research in health: an introduction.

[17] Saunders B, Sim J, Kingstone T (2018). Saturation in qualitative research: exploring its conceptualization and operationalization. Qual Quant.

[18] Gale NK, Heath G, Cameron E (2013). Using the framework method for the analysis of qualitative data in multi-disciplinary health research. BMC Med Res Methodol.

[19] Srivastava A, Thomson SB (2009). Framework analysis: a qualitative methodology for applied policy research. JOAAG.

[20] McSweeney LA, O’Hara JT, Rousseau NS (2017). “Thinking that somebody’s going to delay [a tonsillectomy] for one to two years is quite horrifying really”: a qualitative feasibility study for the NAtional Trial of Tonsillectomy IN Adults (NATTINA Part 2). Clin Otolaryngol.

[21] Stewart MG, Witsell DL, Smith TL (2004). Development and validation of the Nasal Obstruction Symptom Evaluation (NOSE) scale. Otolaryngol Head Neck Surg.

[22] Bellani ML (2008). Psychological aspects in day-case surgery. Int J Surg.

[23] Knops AM, Legemate DA, Goossens A (2013). Decision aids for patients facing a surgical treatment decision: a systematic review and meta-analysis. Ann Surg.

[24] Chisholm EJ, Hajioff D, Kotecha B (2006). Influence of ethnicity on the frequency of nasal surgery. Rhinology.

