# Supplementary material for: Healthcare professionals’ and patients’ views and experiences of surgical and medical treatment for nasal obstruction: a qualitative interview study for a Nasal Airway Obstruction Study (NAIROS)
Source: BMJ Open. 2025 Jun 8;15(6):e099395. doi: 10.1136/bmjopen-2025-099395 (PMC12161377; doi:10.1136/bmjopen-2025-099395)
Supplement: online supplemental file 1 [file bmjopen-15-6-s001.docx]

**Coding framework for NAIROS**

| **Codes** | **Description** |
| --- | --- |
| **Interviewee** | Background info about the participant |
| **Current practice**  Decision-making for septoplasty referral  Patients’ history, characteristics and symptoms  Patients’ prior management  Staffs’ practice pre-NAIROS    Funding for septoplasties | In their current practice, how do staff decide whether to refer a patient for septoplasty  Any description of the patients typically seen in clinic/ on NAIROS (e.g. demographic, any injuries, their symptoms patients typically present with when they are first seen in clinic)  Any way patients have typically tried to manage their symptoms prior to seeing staff in clinic (e.g. medical management from GP)  How staff managed patients’ care before NAIROS (e.g. did they use medical management, did they use the outcome measures before). Also, code here standard practice, e.g. for follow up at this site  Discussion on the general structure and current funding for septoplasties |
| **Views of trial, trial interventions, clinical equipoise**  Quality of evidence for septoplasty  Quality of evidence for medical management  Staffs’ interest in research  Views on medical management vs. surgery  Views on research question  Views on NAIROS protocol  Clinical equipoise  Experience designing the overall trial  Experience designing trial at site  Communal specification  Anticipated impacts of NAIROS trial  Anticipated time constraints  Site accruals | Views on the current evidence for septoplasty  Views on the current evidence for medical management  Staff and colleagues’ general interest in carrying out research/ trials  Views on a trial with a medical management arm and surgery arm, and views on the specific nasal spray vs. septoplasty  Views on the trial’s research question  Views on the trial’s protocol and design  Views on whether the trial has clinical equipoise  If anyone was involved in designing the overall trial, their experiences of this  Experiences of designing the processes of the trial at the site  Sense-making relies on people working together to build a shared understanding of the aims, objectives, and expected benefits of a set of practices. A great example is the team of investigators leading a clinical trial, as they work out how to integrate a complex clinical experiment into a healthcare setting, and as they try to identify and anticipate the relationship between elements of the trial and everyday clinical practice.  Anticipate the results of NAIROS will have on this specialty (e.g. help future or hinder future of septoplasties)  Whether staff have any concerns, prior to starting the trial, on time constraints related to carrying out the trial alongside routine clinical work  How site got on with recruiting patients relative to their target recruitment numbers – including whether feasibility work was overoptimistic |
| **Trial processes**  Site set-up  Screening process  Recruitment process  Consent process  Outcome measures  Specialised research clinics  Views on surgery being offered later  Patients’ treatment preference, agenda, expectations  Patients’ comprehension of randomisation  Patients’ views on their treatment allocation  Patient follow-up  Dropout rates of participants  Crossover of participants  Time constraints during trial  Adherence to inclusion/ exclusion criteria  Impact of NAIROS on clinical work  Priorities during the trial  Technological issues | Staffs’ experiences setting the trial up at their site  Experience of screening participants to take part in the trial  Recruitment of participants to the trial and how they found this  Experience of going through the different consent processes with the participant (e.g. for main trial, for qualitative part, for QRI)  Views and experiences of using the outcome measures in the trial, and pre-NAIROS (e.g. NOSE, SNOT-22)  If they used a specialised research clinic in the trial and their experience of using this  Staff and patients’ views of surgery being offered after the medical management arm  If patients had a preferential treatment and if this affected whether they took part in the trial. Include patient agenda and expectations here  If the patient understood fully the randomisation process  What the patients’ thought of their treatment allocation  How staff found the process of following up patients, and any views on this in general  Any discussion on dropout rates of participants in the trial  Any discussion on participants crossing over from different trial arms  If the staff did experience any time constraints during the trial  Whether the staff adhered to the inclusion/ exclusion criteria of the trial (e.g. did they refer everyone eligible, or exclude certain people)  Whether staff felt NAIROS impacted on their general clinical work and in what ways  The staffs’ main priorities in the trial (e.g. to the research, to the patient)  Any technological issues during the trial, such as the trial database or system for making appointments |
| **Surgical processes**  Organising surgery  Experience of septoplasty  Turbinate reduction  Experience of medical management | Scheduling surgical treatment (8 week window for trial etc.), and other issues around surgery but not the actual craft of the procedure.  How the surgeon conducts septoplasties and their general experience of conducting this operation – the actual craft of the procedure, how they learned to do this procedure etc.  Patients experience of the surgery.  The surgeons’ views on turbinate reduction and whether they do this during surgery  Any discussion on the process and patient and healthcare professionals’ experience of medical management |
| **Community**  Additional support during trial  Communication during the trial  Team effort  Initiation and enrolment  Research nurses  Training for the trial  QRI | Whether staff felt supported during the trial and in what ways  General communication among staff during the trial, either on-site or with central trial team  Any comments from staff on team effort during trial set up and recruitment, including lack of  Detail about leadership at site - who took a lead and how they engaged & encouraged other staff  Views on research nurses role in the trial  Any comments on the training they received for the trial, or any training they would have liked to receive  Any comments on the QRI part of the study |
| **Reflection**  Observed outcomes from trial  Changes in current practice after NAIROS  Changes made to trial process during NAIROS | Expected and unexpected outcomes observed from the trial  Any changes staff have implemented into their current practice as a result of NAIROS, or if they haven’t changed their practice post-NAIROS  Any changes made to trial processes during NAIROS – whether deliberate “to try and improve recruitment we…” or forced by context “we shifted to electronic x so we did this” |
