# Supplementary material for: Healthcare professionals’ and patients’ views and experiences of surgical and medical treatment for nasal obstruction: a qualitative interview study for a Nasal Airway Obstruction Study (NAIROS)
Source: BMJ Open. 2025 Jun 8;15(6):e099395. doi: 10.1136/bmjopen-2025-099395 (PMC12161377; doi:10.1136/bmjopen-2025-099395)
Supplement: online supplemental file 2 [file bmjopen-15-6-s002.docx]

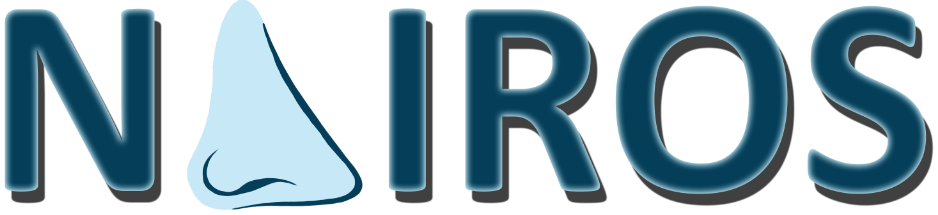


The **N**asal **Air**way **O**bstruction **S**tudy

**NAIROS**

**STAFF INTERVIEW TOPIC GUIDE (Professionals)**

**Version 2.0**

**NAIROS: Interview Topic Guide for Professionals**

***Note:*** *The interview schedule is developmental. The questions will need to be tailored to the specific answers of each interviewee. The interview schedule given here is therefore a general topic guide for the one-to-one qualitative interviews.*

### Welcome and Introduction

Introduce self and project. Discuss confidentiality and recording. Ask if any questions. Confirm ongoing informed consent.

**Re-cap of Research and Plan for Interview**

Brief re-cap on the aims and purpose of the interview and explain what will happen.

***The following questions need not be covered in this particular order but rather the interview should flow as freely and naturally as possible. The interviewer will prompt as appropriate with phrases such as ‘can you tell me a little more about that’, ‘can you give me an example of that’, ‘how did/do you feel about that’.***

### Question: Can you tell me a bit about yourself from the point of view of your career?

### Views on NAIROS

- What point did you hear about it?
- What is your role on the study?
- How do you feel about the research question?
  Is it important?
  What reservations do you have about the NAIROS trial?
- How confident are you that we will be able to recruit to the NAIROS trial?
  *Explore previous experience of surgical trials*

### What has helped you perform your role in NAIROS

- What has made it more difficult?
- How does the NAIROS trial fit with your normal working methods for this patient group?
- How does the NAIROS trial fit with established clinical pathways?
- What impact is recruiting patients for the study having on the day to day running of the clinic?

### Experiences of recruitment

- How do you think recruitment is going so far and how do you go about recruiting patients?
  *Explore previous experience of research and recruitment*
- Have there been any patients who met the inclusion criteria but you felt were not appropriate for the study?
- From your perspective, what are the facilitators and barriers to getting people/patients engaged in the study?
  *Explore facilitators & barriers from staff and patient perspectives*
- Who takes the lead in explaining the study to patients and how do you (they) go about it?
- How else could we support recruitment?

**Rhinospirometry and Peak Nasal Inspiratory Flow (PNIF) meter**

- Were there any issues in setting up the Rhinospirometry or the PNIF meter in this trust?
- Have there been any issues with using the Rhinospirometry or the PNIF meter in this trust?
- Did you find the training provided on the use of the Rhinospirometry and PNIF meter helpful?

### Anything not covered?

### Anything else you’d like to add about your experiences of the study?

**Closing and Thanks**

Conclude the discussion and thank the participant for their time and contribution.
