# Supplementary material for: Healthcare professionals’ and patients’ views and experiences of surgical and medical treatment for nasal obstruction: a qualitative interview study for a Nasal Airway Obstruction Study (NAIROS)
Source: BMJ Open. 2025 Jun 8;15(6):e099395. doi: 10.1136/bmjopen-2025-099395 (PMC12161377; doi:10.1136/bmjopen-2025-099395)
Supplement: online supplemental file 3 [file bmjopen-15-6-s003.docx]

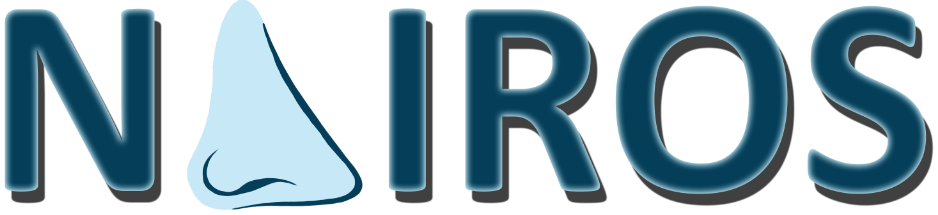


The **N**asal **Air**way **O**bstruction **S**tudy

**NAIROS**

**PATIENT INTERVIEW TOPIC GUIDE**

**Version 2.0**

**NAIROS: Patient Interview Topic Guide**

*Note: The interview schedule is developmental. The questions will need to be tailored to the specific answers of each interviewee. The interview schedule given here is therefore a general topic guide for the one-to-one qualitative interviews.*

**Welcome and Introduction.**

Ask if any questions. Confirm ongoing informed consent

**Re-cap of Research and Plan for Interview**

Brief re-cap on the aims and purpose of the interview and explain what will happen.

***The following questions need not be covered in this particular order but rather the interview should flow as freely and naturally as possible. The interviewer will prompt as appropriate with phrases such as ‘can you tell me a little more about that’, ‘can you give me an example of that’, ‘how did/do you feel about that’.***

**NAIROS recruitment interview questions – participants and decliners**

**I’d like to ask you about being invited to take part in the NAIROS study. Can you tell me how you were first informed about the trial?**

- Who was there?
- What else was going on in the appointment?
- Can you remember what they said?
- Can you remember what your first reaction was?

**How did you decide whether to take part in the study?**

- How did you make your decision?
- What things were important to you?
- Did you speak to other people about the decision? If so, who?
- When did you make the decision?
  - Immediately/nurse phone call/recruitment discussion
- Did you find it an easy decision to make? Why (not)?
- Decliners - how did you feel about saying no?

**Do you remember being given some written information about the study?**

- What did you think about that information?
- Was it helpful?
- How did you use it?
- Do you have any suggestions for how it could be improved? If so, how?

**Would you have liked information about anything else?**

- If so, what?
- Why was that important to you?

**Can you tell me what you understand the study to be about?**

**Follow up interview questions – Trial Participants Only**

**Your experience of the treatments used for a twisted septum**

It is relevant to know what your experience of having a twisted septum (midline nasal partition) was – what sorts of side effects you had and how bad these were. It may be upsetting for the patient to talk about some parts of their experience. It is important to give them time to talk about this. Modify the questions on the basis of what they say about their overall experience.

- Can you tell me about the period of when you had a twisted septum – what was it like?
- How did you find the surgery (if you were randomised to surgery)?
- How did you find the nasal sprays (if you were randomised to Medical Management)?
  - (explore – additional time at appointments, any problems, staff delivering treatment, did they attend all sessions – why/why not?)
- How was your recovery?
- What side effects did you experience?
- How was your breathing after the treatment?
  - (explore – hospital admissions, treatment gaps)
- Did you think the treatment you received worked?
- How have you been since the treatment has finished?
- How are you now?
  - (explore –)

**Anything Not Covered?**

Is there anything that we haven’t covered in the interview that you think we should know or think about?

**Closing and Thanks**

Conclude the discussion and thank the participant for their time and contribution.
