# Supplementary material for: Healthcare professionals’ and patients’ views and experiences of surgical and medical treatment for nasal obstruction: a qualitative interview study for a Nasal Airway Obstruction Study (NAIROS)
Source: BMJ Open. 2025 Jun 8;15(6):e099395. doi: 10.1136/bmjopen-2025-099395 (PMC12161377; doi:10.1136/bmjopen-2025-099395)
Supplement: Supplementary file 4 [file bmjopen-2025-099395.reviewer_comments.pdf]

PEER REVIEW HISTORY

BMJ Open publishes all reviews undertaken for accepted manuscripts. Reviewers are asked to complete a checklist review form and are provided with free text boxes to elaborate on their assessment. These free text comments are reproduced below.

ARTICLE DETAILS

Title (Provisional)

Healthcare professionals' and patients' views and experiences of surgical and medical treatment for nasal obstruction: a qualitative interview study for a nasal airway obstruction trial (NAIROS)

Authors

Lloyd, Kelly E; Wilson, Caroline; Carrie, Sean; Rooshenas, Leila; Wilson, Janet A; Hamilton, David; Clark, Emma; Rousseau, Nikki

---

VERSION 1 - REVIEW

**Reviewer**	**1**
**Name**	**Albazee, Ebraheem**
**Affiliation**	**Kuwait Institute for Medical Specializations**
**Date**	**13-Feb-2025**
**COI**	**None**

Thank you for the opportunity to review the submitted manuscript entitled: "Healthcare professionals' and patients' views and experiences of surgical and medical treatment for nasal obstruction: a qualitative interview study for a nasal airway obstruction trial (NAIROS)"

• This study takes a deep dive into how both healthcare professionals and patients perceive the treatment options for nasal obstruction, specifically comparing septoplasty with medical management using nasal steroid and saline sprays as part of the NAIROS trial. Through interviews with 14 healthcare professionals and 31 patients from different hospitals in the UK, the authors highlight key themes around how treatment decisions are made, patient experiences with each approach, and challenges faced post-treatment. Surgeons often rely on clinical judgment when recommending septoplasty but are open to using standardized tools for better decision-making. Patients' experiences varied—many found septoplasty effective, while others felt it didn't fully resolve their symptoms or came with unexpected side effects. Those who tried medical management had mixed outcomes—some benefited, while others struggled with side effects or found the treatment ineffective.

• The study makes it clear that better patient education and post-treatment guidance are needed to ensure optimal outcomes. While septoplasty generally provided better results,

the findings suggest that medical management can still be a good option for some patients. By improving decision-making tools and shared decision-making processes, both surgeons and patients could feel more confident in choosing the right treatment.

• This is a well-conducted study with real clinical value, and I recommend acceptance as it provides meaningful insights that can directly improve patient care.

**Reviewer**	**2**
**Name**	**Dharanya, Gopalakrishnan**
**Affiliation**	**AIIMS Nagpur, ENT**
**Date**	**23-Feb-2025**
**COI**	**None**

The outcomes of the qualitative research seems to be vaguely defined and has to be precise.

**Reviewer**	**3**
**Name**	**Vennik, Jane**
**Affiliation**	**University of Southamption , Primary care and Population Sciences**
**Date**	**10-Mar-2025**
**COI**	**None**

Abstract

- Could be clearer what 'medical management' is.

- Participants – healthcare professionals – where these just surgeons?

- Conclusion – I would avoid discussing trial results here as this is the qualitative study.

Introduction

- Unclear if medical management was steroid sprays alone or steroid sprays plus saline. Was saline used in the surgical arm?

Design

- This paper has been described as a qualitative process evaluation, but the data presented is nested qualitative work. Please confirm if the trial process evaluation is presented elsewhere? There are alot of other codes/themes in the topic guide.

- Please give details of the qualitative team? Were they independent or part of the trial team?

Participants and recruitment

- Might be helpful to report the location and type of hospital where HCPs were recruited from.

- Data saturation – please describe what you based this on and how you knew that the concept of saturation had been achieved?

- Were the patient and HCP interviews coded separately? How were the findings brought together?

PPIE

- Were the PPIE group involved in interpreting the findings?

Findings

In general – it would be helpful if it were clearer whether the findings were reported by patients only, staff only or patient and staff. At the moment it is difficult to unpick what maps to what group.

- Lines 160-163 – is this what patients said or what staff reported?

- Views on medical management: this section is just based on 2 accounts (research nurse and surgeon). I'm not sure this is sufficient to warrant a sub-theme.

- Line 291 – should this be uses the sprays/rinses rather than take the medicine?

- Line 291 – what was the evidence that using the sprays was 'burdonsome'? Did patients say?

- Line 293 – some patients reported side effects. What were these (other than nose bleeds?).

- Were patients given any information about how to use the sprays?

Discussion

The first paragraph of the discussion is about the process evaluation of the trial, but the following details are about making decisions about treatment (deciding who should have medical or surgical management). In the trial, patients would be randomly allocated to a treatment arm. I think it should be made clearer that this paper is either about the trial or the implementation of the trial findings. At the moment it is a bit of a combination.

Line 334 I'm not sure that you can claim that the qualitative work demonstrated better symptom management in the surgical compared to the medical arm. Qualitative work uses purposeful sampling and is to obtain a range of views and experiences, and is not powered to compare between groups.

Line 356 – Patients did not receive instruction for the sprays – this should be in the findings section. First time mentioned here.

Strengths and limitations

- Is there any evidence that ethnicity may affect how people experience and manage septoplasty? Using nasal sprays?

- Are trial participants more likely to engage in self-management behaviours such as using nasal sprays?

VERSION 1 - AUTHOR RESPONSE

Reviewer: 1

Dr. Ebraheem Albazee, Kuwait Institute for Medical Specializations

Comments to the Author:

Thank you for the opportunity to review the submitted manuscript entitled: “Healthcare professionals’ and patients’ views and experiences of surgical and medical treatment for nasal obstruction: a qualitative interview study for a nasal airway obstruction trial (NAIROS)”

This study takes a deep dive into how both healthcare professionals and patients perceive the treatment options for nasal obstruction, specifically comparing septoplasty with medical management using nasal steroid and saline sprays as part of the NAIROS trial. Through interviews with 14 healthcare professionals and 31 patients from different hospitals in the UK, the authors highlight key themes around how treatment decisions are made, patient experiences with each approach, and challenges faced post-treatment. Surgeons often rely on clinical judgment when recommending septoplasty but are open to using standardized tools for better decision-making. Patients' experiences varied—many found septoplasty effective, while others felt it didn’t fully resolve their symptoms or came with unexpected side effects. Those who tried medical management had mixed outcomes—some benefited, while others struggled with side effects or found the treatment ineffective.

The study makes it clear that better patient education and post-treatment guidance are needed to ensure optimal outcomes. While septoplasty generally provided better results, the findings suggest that medical management can still be a good option for some patients. By improving decision-making tools and shared decision-making processes, both surgeons and patients could feel more confident in choosing the right treatment.

This is a well-conducted study with real clinical value, and I recommend acceptance as it provides meaningful insights that can directly improve patient care.

Authors' reply: Thank you for taking the time to review our manuscript and for your kind comments.

Reviewer: 2

Dr. Gopalakrishnan Dharanya, AIIMS Nagpur

Comments to the Author:

The outcomes of the qualitative research seems to be vaguely defined and has to be precise.

Authors' reply: Thank you for reviewing our manuscript. We have amended our aims of the paper to be clearer at the beginning of the methods section and for these to link better to our outcomes, which are the themes in the paper. Please see the amendment below:

> *We conducted a nested qualitative study to explore the experiences of patients and clinicians participating in the NAIROS trial. We aimed to capture healthcare professionals' and patients' views and decision-making on septoplasty and medical management, and their experiences of delivering or undergoing these treatments. (Methods, page 5, lines 101-04).*

Reviewer: 3

Dr. Jane Vennik, University of Southampton

Comments to the Author:

Abstract

- Could be clearer what 'medical management' is.
- Participants – healthcare professionals – where these just surgeons?
- Conclusion – I would avoid discussing trial results here as this is the qualitative study.

Authors' reply: Thank you for reviewing our manuscript. We have amended our abstract to be clearer what medical management is:

> ***Objectives:*** *To understand healthcare professionals' and patients' views and experiences of septoplasty and medical management (i.e., nasal steroid and saline sprays) for nasal obstruction.*

Healthcare professional participants included both surgeons and nurses, which we have clarified in the Abstract:

> ***Participants:*** *We purposively sampled and interviewed 14 healthcare professionals (surgeons, research nurses) and 31 patients involved in the NAIROS trial across 14 UK hospital sites.*

We have also removed discussion of the trial results from the abstract.

> ***Conclusion:*** *Our qualitative study demonstrated varied individual experiences among patients undergoing septoplasty and medical management. Surgeons welcomed more standard measurements to guide decision-making for septoplasty. For patients, better information about treatment mechanisms, treatment delivery and aftercare, and the development of decision support tools would enable shared decision making, and help to provide optimal patient experience of the treatments.*

Comments to the Author: Introduction
- Unclear if medical management was steroid sprays alone or steroid sprays plus saline. Was saline used in the surgical arm?

Authors' reply: We have made clearer in the Abstract and in the Introduction that medical management included both the steroid and saline sprays, which was compared to septoplasty.

> ***Objectives:*** *To understand healthcare professionals' and patients' views and experiences of septoplasty and medical management (i.e., nasal steroid and saline sprays) for nasal obstruction. (Abstract)*
>
> *Patients were randomised to either septoplasty or daily nasal steroid and saline sprays. (Introduction, page 4, lines 90 to 91).*

Comments to the Author: Design
- This paper has been described as a qualitative process evaluation, but the data presented is nested qualitative work. Please confirm if the trial process evaluation is presented elsewhere? There are a lot of other codes/themes in the topic guide.

Authors' reply: Thank you for highlighting this. The data describing the qualitative trial processes will be published in a separate paper.

We have changed reference of the qualitative process evaluation in our manuscript to a nested qualitative study, e.g.:

> ***Design:*** *Nested qualitative study as part of the Nasal Airway Obstruction Study (NAIROS) trial. We used in-depth interviews to develop a coding framework based on thematic analysis. (Abstract).*
>
> *A qualitative nested study was also conducted alongside the trial. (Introduction, page 4, lines 92 to 93).*
>
> *We conducted a nested qualitative study to explore the experiences of patients and clinicians participating in the NAIROS trial. (Methods, page 5, lines 101 to 102).*

Comments to the Author: Please give details of the qualitative team? Were they independent or part of the trial team?

Authors' reply: We have now included further detail on this.

> *The topic guide was updated during the study on the basis of early interviews and participation in the NAIROS trial management group. Interviews were conducted by three experienced qualitative researchers (JM, LL and CW). NR, JM, LL and CW were involved in the nested qualitative study and were part of the NAIROS trial team and attended the trial management group meetings. (Methods, page 5, lines 127 to 131)*
>
> *One researcher (KL) coded all staff and patient interviews using this single coding framework. KL joined the qualitative team during trial follow up and analysis and was not part of the NAIROS trial team. (Methods, page 6, lines 145 to 147)*

Comments to the Author: Participants and recruitment
- Might be helpful to report the location and type of hospital where HCPs were recruited from.

Authors' reply: To protect participants' identity, we have anonymised the hospital locations in our paper. We have now made it clear in our paper that these were 17 secondary care hospitals located in England, Scotland and Wales, and that the hospitals are listed in the full NIHR report.

> *The trial was conducted across 17 secondary care hospital trusts in England, Scotland and Wales, with the locations listed in the full trial report[10]. (Introduction, page 4, lines 89 to 90).*

Comments to the Author: Data saturation – please describe what you based this on and how you knew that the concept of saturation had been achieved?

Authors' reply: We have now included additional detail on our data saturation approach:

> *During data collection, regular meetings were held (NR and JM/CW) to review transcripts, discuss the preliminary findings and to make decisions regarding further data collection[16]. Recruitment ceased when it was agreed at these meetings that we had collected rich and comprehensive data regarding the topics under investigation, that recent data collection was adding relatively few new insights, and that further data collection was unlikely to add substantially to the analysis, based on the preliminary analysis and with reference to the purposive criteria (data saturation)[17]. (Method, page 6, lines 136 to 140)*

Comments to the Author: Were the patient and HCP interviews coded separately? How were the findings brought together?

Authors' reply: Thank you for highlighting this. We have now made our process clearer for how we analysed the patient and healthcare professional interviews together:

> *Two researchers (NR and KL) developed the framework after reading through several transcripts, and after reviewing previous literature on qualitative research in trials (e.g., [20]). A single coding framework was developed for both patient and staff interviews (supplementary materials), as many codes were deemed to be relevant to both participant groups (e.g., both groups described their experiences of septoplasty and medical management). One researcher (KL) coded all staff and patient interviews using this single coding framework. KL joined the qualitative team during trial follow up and analysis and was not part of the NAIROS trial team. Two authors (KL and NR) discussed the codes collaboratively and generated themes through discussions. (Methods, page 6, lines 140 to 148).*

Comments to the Author: PPIE
- Were the PPIE group involved in interpreting the findings?

Authors' reply: The PPIE group were involved in the design of the qualitative, which we have reported on page 6. They were not involved in the analysis.

Comments to the Author: Findings

In general – it would be helpful if it were clearer whether the findings were reported by patients only, staff only or patient and staff. At the moment it is difficult to unpick what maps to what group.

- Lines 160-163 – is this what patients said or what staff reported?

Authors' reply: We have amended the text in the results to make clearer in places, including the suggestion above, the data reported by patients, staff, and by both groups. E.g.:

> *Both patients and staff reported that nasal obstruction resulted in a wide range of symptoms. Patients described presenting with a range of different symptoms in clinic including nasal blockage, trouble sleeping, and frequent nosebleeds (Table 3). Several patients could recall an injury that had damaged or broken their nose, while others did not remember such an incident. Staff members also reported a complex pattern and history of symptoms presented by patients in clinic. (Results, page 7, lines 171 to 175).*
>
> *While surgeons often recommended medical management first, some patients interviewed described how they could not always see the value of medical management first, as they perceived the cause of their symptoms to be from a traumatic injury that only surgery could "fix". (Results, page 8, lines 201 to 203).*

Comments to the Author: Views on medical management: this section is just based on 2 accounts (research nurse and surgeon). I'm not sure this is sufficient to warrant a sub-theme.

Authors' reply: Thank you for highlighting this. We have now expanded upon this sub-theme with the below text and quotes (Results, pages 10 to 11):

> *Similar to the research nurse, this surgeon felt that their views on medical management had changed as a result of participating in NAIROS, although they remained sceptical as to whether it was a viable long-term solution for patients.*
>
> *"Yeah I think they have [views on medical management have changed]. I think in some respect I've been surprised about some patients being quite positive about it. ... I think on balance for the 12 months that they're taking the medication, some of them have certainly found it useful. I'm not sure how that would play out if we'd sort of monitored them all over five years or so." Surgeon 7*

Whether this change in view of the effectiveness of medical management would result in changes in practice was less clear. Most surgeons reported that they already often recommended steroid sprays to patients before offering surgery. However routine recommendation of saline sprays had been relatively uncommon prior to NAIROS, with one surgeon reporting that as a result of their participation in the NAIROS trial they now recommended the use of saline sprays to patients.

"Personally, I don't think it's changed what I do. I've always maintained giving them proper medical treatment first." Surgeon 6

"I didn't tend to use the saline douching as much as the protocol did [...]. I tend to now, because I sort of got used to the study protocol and I think, 'Yes, that makes some sense'." Surgeon 2

Comments to the Author: Line 291 – should this be uses the sprays/rinses rather than take the medicine?

Authors' reply: Thank you, we have amended this.

Comments to the Author: Line 291 – what was the evidence that using the sprays was 'burdonsome'? Did patients say?

Authors' reply: Patients didn't explicitly use the word burden – but several of them did discuss struggling to remember to use the sprays and about the need to set up routines to help with this. We have interpreted their data to mean that remembering to use the sprays can be 'burdensome'. We have now expanded on this and included a quote to evidence this to the paper:

For patients in the medical management group, remembering to use the sprays could be burdensome. Patients sometimes described establishing a routine to help them remember, for example placing the bottle next to their toothpaste.

*"Using them has been no problem at all. I think it's remembering when to use them and the frequencies to use. Initially, I had to keep checking on the packaging and what have you. But, as time has gone on, **I've found it a lot easier,** and it's one of the first- I just do it in the morning now, so I know exactly when I'm having it." Patient 20 (Results, page 12 lines 317 to 323)*

Comments to the Author: Line 293 – some patients reported side effects. What were these (other than nose bleeds?).

Authors' reply: We have now clarified that another side-effect was pain:

> *There was a lack of clear information on how patients should administer the sprays to an obstructed nasal passage, which resulted in some patients experiencing side-effects, such as nosebleeds and pain. (Results, page 12, lines 324 to 326)*

There is an existing quote as well where a participant discusses experiencing painful side-effects using the sprays:

> *"They asked me if I'd want to carry on for a little bit longer with the nasal spray, so I tried for another week,* ***but it was just too sore****. [...] I didn't really see any changes at all, not for the better and not for the worse either." Patient 1 (Results, page 12, lines 337 to 339)*

Comments to the Author: Were patients given any information about how to use the sprays?

Authors' reply: Thank you for highlighting this, we have now clarified this in the Results section:

> *There was a lack of clear information on how patients should administer the sprays to an obstructed nasal passage, which resulted in some patients experiencing side-effects, such as nosebleeds and pain. One participant reported being advised that their nosebleeds may have been associated with the mode of administration (e.g., inserting the spray bottle too deeply), and other people highlighted that using the spray was difficult when there was an obstruction. (Results, page 12, lines 324 to 328)*

Comments to the Author: Discussion
The first paragraph of the discussion is about the process evaluation of the trial, but the following details are about making decisions about treatment (deciding who should have medical or surgical management). In the trial, patients would be randomly allocated to a treatment arm. I think it should be made clearer that this paper is either about the trial or the implementation of the trial findings. At the moment it is a bit of a combination.

Authors' reply: Thank you for highlighting this. We have now replaced parts of the paper which described the paper as a qualitative process evaluation, and have changed this to nested

qualitative study. We have also made clearer in the Design section of the Methods the aims of this paper, which are aligned with the findings of our paper and the Discussion section:

> *We conducted a nested qualitative study to explore the experiences of patients and clinicians participating in the NAIROS trial. We aimed to capture healthcare professionals' and patients' views and decision-making on septoplasty and medical management, and their experiences of delivering or undergoing these treatments. (Methods, page 5, lines 101-04).*

We have also amended the first line of the Discussion:

> *Our nested qualitative study conducted as part of the NAIROS trial identified considerable variation and complexity in decision-making about septoplasty. (Discussion, page 13, lines 353 to 354)*

We have also noted that a limitation of our study is that the NAIROS trial allocated participants to their treatment:

> *While we aimed to explore decision-making on septoplasty and medical management, participants were randomly assigned to their treatment as part of the NAIROS trial. However, we also explored participants' wider views, including surgeons' decision-making on septoplasty in routine care. (Discussion, page 15, lines 408 to 411)*

Comments to the Author: Line 334 I'm not sure that you can claim that the qualitative work demonstrated better symptom management in the surgical compared to the medical arm. Qualitative work uses purposeful sampling and is to obtain a range of views and experiences, and is not powered to compare between groups.

Authors' reply: We have now amended this to the following:

> *In our interview study, we observed differing levels of symptom resolution of nasal obstruction among patients in both medical and surgical management arms, following receipt of their allocated treatment. However, many patients interviewed who underwent septoplasty reported experiencing symptom reduction, which is aligned with the NAIROS clinical trial results. (Discussion, page 13, lines 365 to 366)*

Comments to the Author: Line 356 – Patients did not receive instruction for the sprays – this should be in the findings section. First time mentioned here.

Authors' reply: Thank you for highlighting this, as noted above we have now included the following in the Results section:

> *There was a lack of clear information on how patients should administer the sprays to an obstructed nasal passage, which resulted in some patients experiencing side-effects, such as nosebleeds and pain. (Results, page 12, lines 324 to 326)*

Comments to the Author: Strengths and limitations
- Is there any evidence that ethnicity may affect how people experience and manage septoplasty? Using nasal sprays?

Authors' reply: Thank you for noting this, we have now expanded upon this limitation with evidence in this area:

> *The patient sample were also limited on certain demographics, with a majority white sample. This is important because there is evidence of variation in the frequency of surgery for nasal obstruction across different ethnic groups, with people who identify as Chinese or Black less likely to undergo this surgery than people who identify as white[24]. Further research is needed to understand this variation. (Discussion, page 15, lines 411 to 415)*

- Are trial participants more likely to engage in self-management behaviours such as using nasal sprays?

Authors' reply: We have now included this as a limitation in our paper:

> *A limitation is that we interviewed staff and patients recruited to a clinical trial, which may create difficulties when generalising the findings to routine care. In particular, patients who took part in the trial may be more motivated to engage with and adhere to the sprays, compared with those typically seen in routine care. (Discussion, page 14 to 15, lines 406 to 408).*

VERSION 2 - REVIEW

**Reviewer**	**3**
**Name**	**Vennik, Jane**

Affiliation **University of Southamption , Primary care and Population Sciences**

Date **19-May-2025**

COI

The authors have addressed all of my concerns and comments.
